# Lectures on the Principles and Practice of Physic, Delivered at King’s College, London

**Published:** 1847-12

**Authors:** 


*Lectures on the Principles and Practice of Physic, delivered at King's College, London.* By Thomas Watson, M. D., Fellow of the Royal College of Physicians; late Physician to the Middlesex Hospital, &c. Third American, from the last London edition. Revised, with additions. By D. Francis Condie, M. D., Secretary of the College of Physicians; Author of a Treatise on Diseases of Children, etc., etc. Philadelphia: Lea & Blanchard. 1847. 8vo. p.p. 1040.

If the sentiments of those with whom we are acquainted, furnish any criterion by which to measure the current opinion of the profession, in point of present popularity, Dr. Watson's work bears the palm. No other work on practical medicine, at this moment, is more highly and generally esteemed. This could hardly be the case if the work did not possess sterling excellencies. It is, however, in some measure due to the colloquial, off-hand style in which it is written. The author candidly states that the lectures were hastily prepared, and, from internal evidences, it is easy to perceive that they were written *currente calamo*. But for this very reason they are more attractive. Had he devoted ample leisure and meditation to the composition, he might, perhaps, have made a better work; but we much doubt if he would have succeeded so well in those qualities which engage the attention and interest of the reader. Published lectures, in which an easy, conversational style is happily preserved, are the most acceptable form in which scientific instruction can be communicated by books, because it possesses, in some measure, the advantages which appertain to oral teaching. We seem to be brought into nearer personal relations with the author, and his writings have a practical cast which we cannot so well appreciate in more formal and elaborate productions.

Giving our opinion frankly for what it is worth, we deem the late work by Dr. Wood a better text book than that of Dr. Watson; but, for the reasons just mentioned, it is, perhaps, less attractive. In making this remark, however, we have no intention to disparage the merits of the latter. Dr. Watson is not only an agreeable writer, but a sensible, judicious, well-informed practitioner. We believe it is not doing injustice to medicine *as it now is*, to say that the work is a faithful exponent of its principles and practice.

The reader will perceive, by the number of its pages, that it embraces a large amount of matter. It has the additional recommendation of being furnished at a very reasonable price.

Dr. Condie's additions denote judgment and good taste; and for the American reader enhance the value of the work.

The *getting up* is excellent.

